# Quantitative Visualization of Intracellular Nanometabolism Using Peak‐Shifted Dual‐State Emissive FRET Nanoprobes

**DOI:** 10.1002/advs.77509

**Published:** 2026-09-06

**Authors:** Farsai Taemaitree, Jinwoo Sung, Yutaro Miki, Ryuju Suzuki, Yoshitaka Koseki, Kunikazu Ishii, Minsang Kim, Keita Tanita, Kota Sato, Toru Nakazawa, Satoshi Katsube, Daisuke Unabara, Tasuku Hamaguchi, Keisuke Kawakami, Koji Yonekura, Akihide Hibara, Atsushi Wakamiya, Hiroshi Uji‐I, Hitoshi Kasai

**Affiliations:** ^1^ Research Institute for Electronic Science and Division of Information Science and Technology Hokkaido University Sapporo Japan; ^2^ Institute of Multidisciplinary Research for Advanced Materials Tohoku University Sendai Japan; ^3^ National Institute of Technology Sendai College Sendai Japan; ^4^ Department of Life and Environmental Science Prefectural University of Hiroshima Hiroshima Japan; ^5^ Department of Ophthalmology Tohoku University Sendai Japan; ^6^ Department of Advanced Ophthalmic Medicine Tohoku University Sendai Japan; ^7^ Department of Retinal Disease Control Tohoku University Sendai Japan; ^8^ RIKEN SPring‐8 Center Sayo Japan; ^9^ Department of Chemistry Institute of Science Tokyo Tokyo Japan; ^10^ Institute for Chemical Research Kyoto University Kyoto Japan; ^11^ Division of Molecular Imaging and Photonics Department of Chemistry KU Leuven Leuven Belgium; ^12^ Institute for Integrated Cell‐Material Science (WPI‐iCeMS) Kyoto University Kyoto Japan

**Keywords:** bioimaging, dual‐state emission, metabolism, nanomedicine, nanoparticles

## Abstract

Many nanoscale materials, ranging from environmental particulates to carrier‐free nanomedicine platforms, enter the human body, yet their metabolic fates remain poorly understood. This process involves complex physical changes and molecular transformations within lysosomes; however, the particle‐to‐molecule transition–defined here as “nanometabolism”–has remained difficult to quantify due to intrinsic limitations of nanoscale bioimaging. To circumvent the fluorescence quenching of conventional fluorophores and the challenges of observing nanoscale dynamics, peak‐shifted, dual‐state emissive nanoprobes composed entirely of donor–acceptor bithiophene dyes are introduced here. This approach establishes a highly adaptable design strategy for constructing heterogeneous organic nanoparticles templated by these molecular metrics. These molecular probes generate bright, ratiometric spectral shifts that directly encode nanoparticle disassembly, enabling robust optical monitoring of intracellular nanometabolism with cell‐type‐ and particle‐size‐resolution. Time‐resolved imaging identifies protonation‐induced lysosomal membrane destabilization as the primary driver of nanoparticle breakdown within 6–12 h. Supported by cryogenic correlative imaging and tissue‐level tracking, this platform provides a promising optical framework for monitoring nanometabolism in living systems. It serves as a pivotal breakthrough in elucidating the mechanistic design rules required for the future development of carrier‐free nanomedicines with predictable intracellular behavior.

## Graphical abstract

This study reveals the long‐elusive intracellular dissolution mechanism of carrier‐free nanomedicines. By utilizing peak‐shifted dual‐state emissive FRET nanoprobes (PDFNPs) that undergo a distinct ratiometric fluorescence peak shift upon disassembly, we achieve real‐time, quantitative tracking of dissolution kinetics in live cells. This sensing framework provides a robust foundation for designing next‐generation carrier‐free nanotherapeutics.

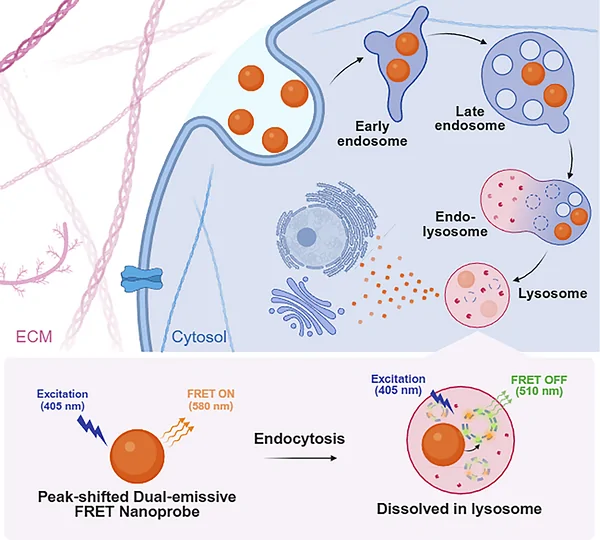

## 1 Introduction

Organic nanoparticles are gaining significant traction as platforms for carrier‐free nanomedicines due to their exceptional drug‐loading capacity and the absence of carrier‐associated toxicity [1, 2, 3, 4, 5, 6]. Beyond therapeutic applications, humans are routinely exposed to environmental organic particulates; however, the intracellular fate of these poorly soluble nanoparticles remains similarly poorly understood [7, 8, 9, 10, 11, 12]. We specifically define the critical transition from an assembled particulate state to a dissolved molecular state within lysosomes as “intracellular nanometabolism.” This fundamental transformation dictates therapeutic efficacy and biological outcomes, yet it has remained inaccessible to precise optical interrogation. Carrier‐free nanomedicines, typically assembled directly from hydrophobic drug molecules [13, 14, 15, 16], epitomize this challenge. Their inherent hydrophobicity confers resistance to premature enzymatic degradation, facilitating intact cellular internalization [17, 18]; however, pharmacological activation is strictly contingent upon this particle‐to‐molecule nanometabolism, underscoring the urgent need to elucidate their lysosomal dissolution kinetics and pathways.

**FIGURE 1:**
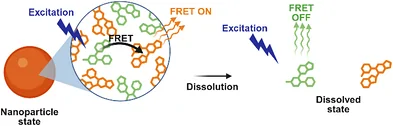
Ratiometric FRET strategy for monitoring the nanoparticle‐to‐molecular phase transition using dual‐state emissive fluorophores. Visualizing intracellular nanometabolism requires a continuous optical narrative spanning the entire solid‐to‐molecular phase transition. Conventional fluorophores, however, possess inherent optical blind spots: ACQ dyes are fluorescently silent in the initial aggregate state, while AIE dyes lose their signal upon molecular dissolution. To overcome these binary limitations, a ratiometric FRET strategy is employed, where the structural collapse of the nanoparticle triggers a distinct spectral shift from a FRET‐ON (particulate) to a FRET‐OFF (dissolved) state. The success of this transition relies on the use of dual‐state emissive (DSE) fluorophores, which, unlike traditional dyes, maintain high radiative transition rates in both aggregated and monomeric forms. This unique photophysical trait ensures uninterrupted fluorescence signaling throughout the dissolution process, enabling the precise spatiotemporal tracking of the nanodrug surrogate's metabolic fate.

A major obstacle in monitoring this process has been the inherent limitations of conventional fluorescence imaging. Most organic fluorophores are categorized as either Aggregation‐Caused Quenching (ACQ) or Aggregation‐Induced Emission (AIE) materials, both of which possess significant optical blind spots. ACQ fluorophores undergo non‐radiative decay in the aggregated state, rendering the initial nanoparticle state non‐fluorescent (OFF). Conversely, AIE fluorophores become non‐fluorescent (OFF) upon molecular dissolution. Because both systems lose their signal at one end of the metabolic lifecycle, they are fundamentally incapable of providing a continuous, real‐time narrative of the particle‐to‐molecular phase transition. To overcome the binary nature of single‐fluorophore systems, Förster Resonance Energy Transfer (FRET) has been utilized to distinguish closely packed nanoparticles (FRET‐ON) from dispersed molecules (FRET‐OFF). However, the development of reliable FRET nanoprobes is hindered by a fundamental challenge: the stringent requirement for dual‐state emission. Both the donor and acceptor must maintain high quantum yields during the transition from aggregate to monomer—a photophysical trait rarely observed in conventional dyes (Figure 1).

Here, we introduce peak‐shifted dual‐state emissive FRET nanoprobes (PDFNPs) to bypass these fundamental constraints. Composed entirely of structurally analogous donor–acceptor bithiophene dyes (BBTP [19]), PDFNPs leverage high molecular affinity to prevent phase separation while maintaining bright, dual‐state emission. This small‐molecule‐based architecture permits unrestricted donor–acceptor stoichiometry, establishing a generalizable design principle for heterogeneous organic nanoparticles.

PDFNPs provide direct, quantitative optical readouts of their structural integrity through ratiometric peak shifts, outperforming conventional ON/OFF‐type probes in both sensitivity and robustness. By establishing a highly sensitive optical window into intracellular nanometabolism, this platform serves as a powerful proof‐of‐concept methodology for tracking cell‐type‐ and particle‐size‐dependent lysosomal processing with unprecedented clarity. Ultimately, this dual‐state emissive strategy represents a pivotal breakthrough in elucidating the mechanistic design rules that guide the rational development of next‐generation, carrier‐free organic nanotherapeutics with predictable intracellular behavior.

## 2 Results and Discussion

### 2.1 Design and Characterization of Peak‐Shifted Dual‐State Emissive FRET Nanoprobes (PDFNPs)

To elucidate the mechanisms governing nanometabolism, we engineered a highly emissive nanoprobe architecture that maintains robust fluorescence in both particulate and dissolved states. We utilized a bithiophene core functionalized with a bulky dimesitylboryl group to suppress detrimental intermolecular π–π interactions, thereby mitigating aggregation‐caused quenching (ACQ). The 3‐boryl‐2,2’‐bithiophene (BBTP) framework provides two strategic advantages for tracking dissolution kinetics: the sterically hindered boryl group and large Stokes shifts—arising from intramolecular charge transfer (ICT)—ensure a high fluorescence quantum yield (ΦF) even in the aggregated state; and the modularity of the core allows for fine‐tuning the emissive properties arranged for FRET across a broad spectral range (477–600 nm) [19]. Crucially, this spectral tunability enables precise engineering of FRET pairs by optimizing the spectral overlap between donor emission and acceptor absorption, a prerequisite for maximizing energy‐transfer efficiency within the organic nanoparticle matrix.

**FIGURE 2:**
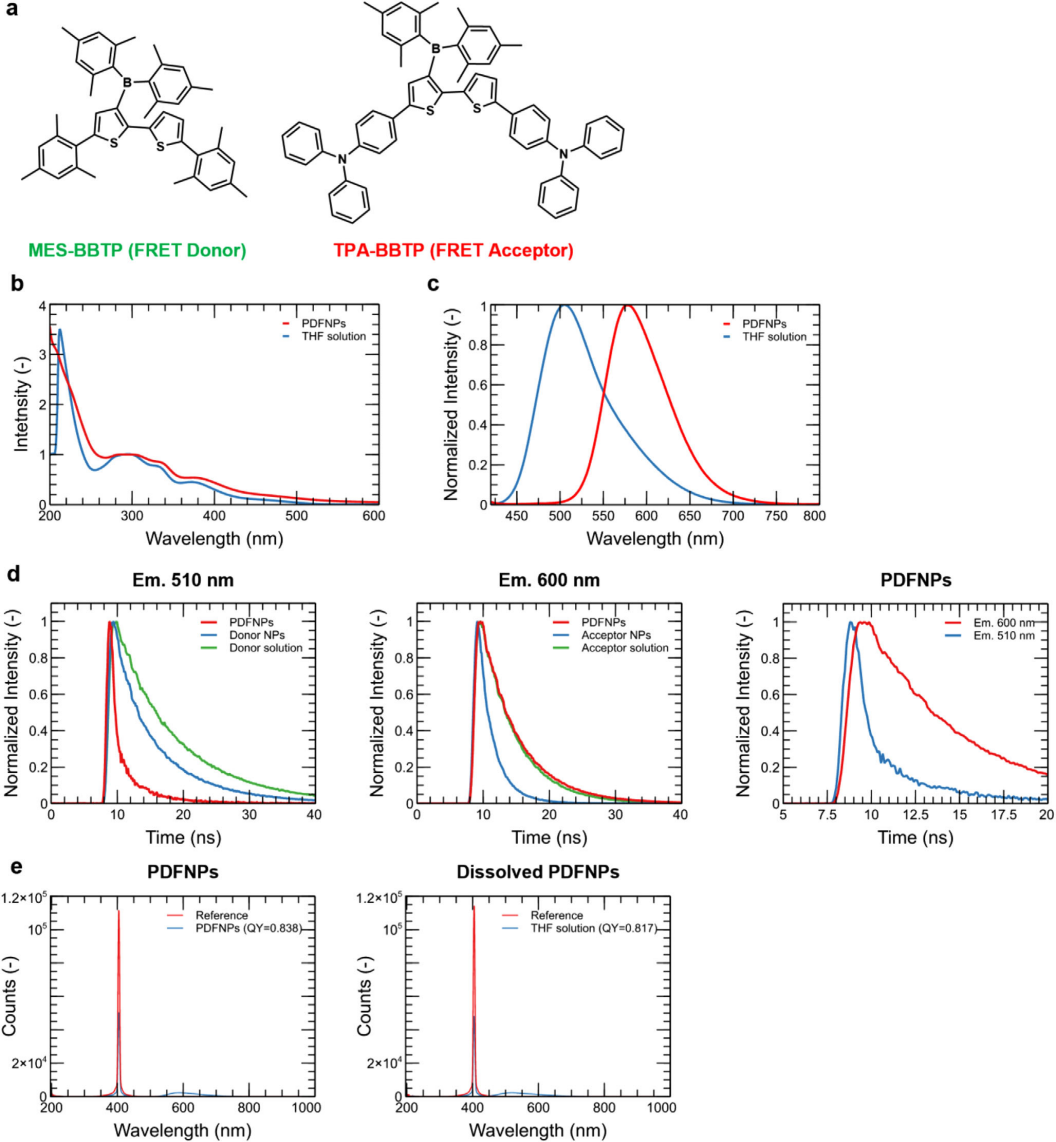
Design principle and photophysical characterization of dual‐emissive FRET nanoprobes (PDFNPs). (a) Chemical structures of TPA‐BBTP (donor) and MES‐BBTP (acceptor). (b) Absorption spectra of PDFNPs in the nanoparticulate and dissolved states (in THF). (c) Fluorescence emission spectra (λex = 405 nm) highlighting the significant ratiometric peak shift triggered by PDFNP dissolution. (d) Time‐resolved fluorescence lifetime analysis confirming the FRET process. The accelerated decay of the donor‐derived signal (510 nm) and the delayed rise/extended lifetime of the acceptor‐derived signal (600 nm) in the PDFNP state validate efficient intra‐particle energy transfer. (e) Comparison of fluorescence quantum yields (ΦF) demonstrating the robust emissive properties of PDFNPs in both solid and molecular phases.

From a library of BBTP derivatives, MES‐BBTP and TPA‐BBTP were selected as the FRET donor and acceptor, respectively, due to their optimized spectral overlap (Figure 2A). Both fluorophores can be excited at a single wavelength (405 nm) yet possess distinct emission profiles, a critical feature for ratiometric sensing. We fabricated a series of nanoprobes with varying donor‐to‐acceptor ratios and identified the 90:10 ratio as the optimal composition, yielding the most pronounced ratiometric spectral shift upon dissolution (Figure S1).

A dramatic spectral migration upon the particle‐to‐molecule transition characterized the optical signature of the PDFNPs. Notably, no significant peak shifts or sharpening were observed in the absorption spectra (Figure 2B) before and after nanoparticulation, showing an exceptionally high degree of spectral coincidence. This preservation of the absorption profile strongly suggests that the encapsulated dyes do not form highly ordered, crystalline supramolecular arrangements, such as H‐ or J‐aggregates. Instead, while the absorption spectra remained nearly identical, the fluorescence emission peak shifted from 580 nm (FRET‐ON state in nanoparticles) to 500 nm (FRET‐OFF state in monomeric solution) (Figure 2B,C). This 80 nm shift provides a high‐contrast optical readout for monitoring nanometabolism. Furthermore, the disordered nature of the internal packing was directly corroborated by the X‐ray diffraction (XRD) pattern (Figure S2E), confirming that the PDFNPs possess a predominantly amorphous internal architecture.

To validate the FRET mechanism, we performed time‐resolved fluorescence spectroscopy. In the PDFNP state, the donor lifetime was significantly shorter (3.5 ns) than in the donor‐only control (7.4 ns). In comparison, the acceptor exhibited a characteristic delayed rise and prolonged decay (5.6 ns vs. 2.4 ns for acceptor‐only) (Figure 2D). These results confirm efficient long‐range dipole‐dipole energy transfer within the densely packed nanoparticle matrix. PDFNPs exhibited a high ΦF of 0.84 in the nanoparticle state and 0.82 upon dissolution (Figure 2E). This remarkable consistency in quantum yield between solid and dissolved phases—addressing the optical blind spots of ACQ/AIE materials—confirms that PDFNPs are uniquely suited for the quantitative and continuous tracking of intracellular nanometabolism.

To further corroborate that the observed ratiometric shift originates exclusively from intra‐particle FRET rather than from nonspecific interparticle interactions, we conducted a series of control experiments using single‐component nanoprobes. First, we confirmed that the intrinsic fluorescence profiles of donor‐only and acceptor‐only nanoprobes remained unchanged upon nanoparticulation (Figure S2B,C). These individual nanoprobes were then mixed in the same stoichiometric ratio as the PDFNPs to create a dispersed system of spatially separated donor and acceptor particles. Consequently, unlike the formulation‐optimized PDFNPs, this physical mixture of single‐component nanoprobes—adjusted to identical concentrations and compositions—exhibited a dominant donor‐derived fluorescence emission peak (Figure S2D). This distinct optical contrast provides definitive validation that the ratiometric spectral migration observed in PDFNPs is explicitly driven by high‐fidelity FRET between the homogeneously co‐packed donor and acceptor molecules within a single nanostructure.

**FIGURE 3:**
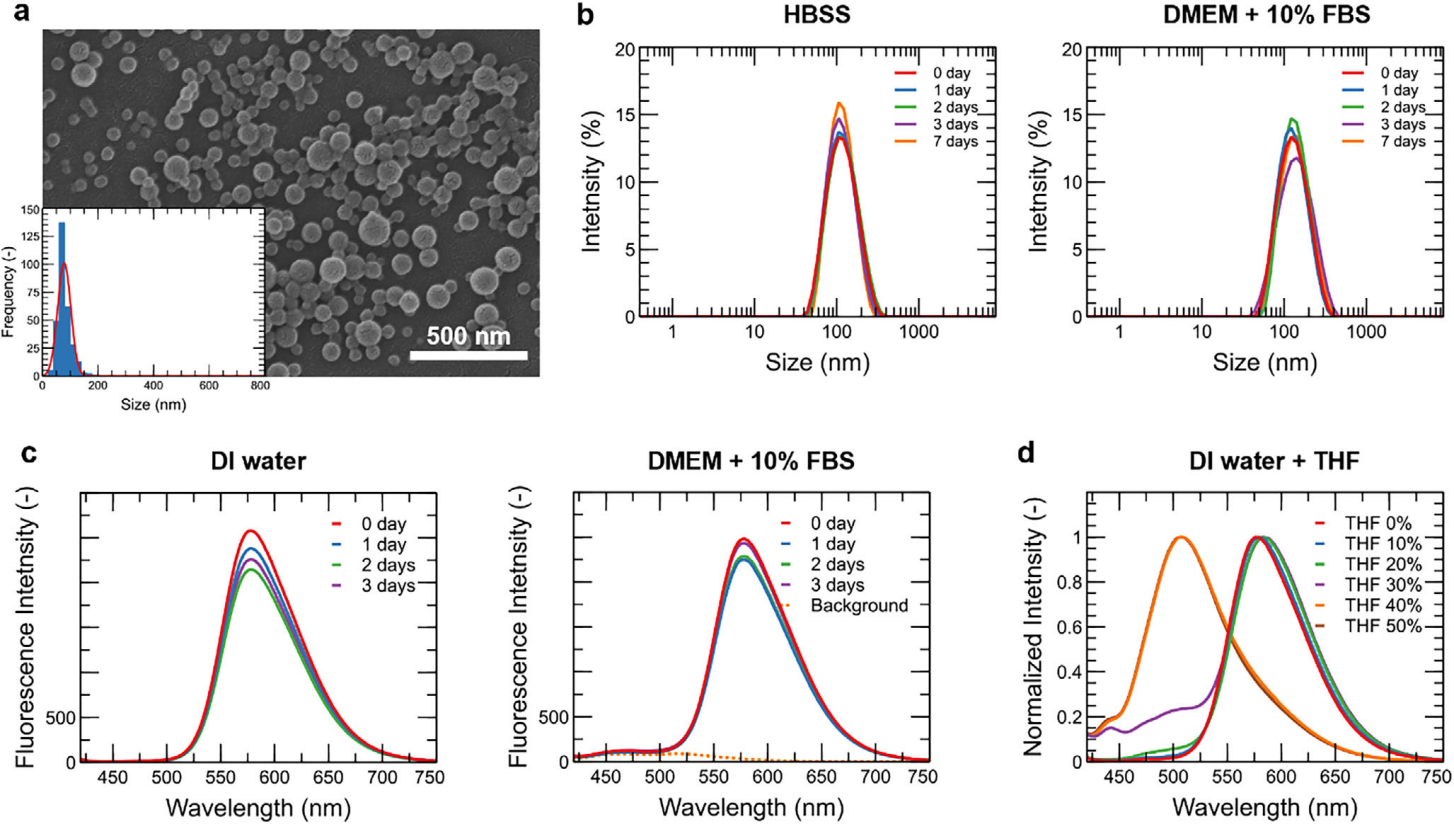
Morphological characterization and environment‐responsive optical signatures of PDFNPs. (a) Representative SEM image illustrating the formation of monodisperse PDFNPs with diameters <100 nm (Scale bar: 500 nm). Size distribution histogram of PDFNPs fitted with a Gaussian curve (n = 300), demonstrating their highly uniform size and monodispersity. (b) DLS profiles showing the size distribution and exceptional colloidal stability in protein‐free (left) and protein‐rich (right) media over 7 days. (c) Time‐resolved fluorescence spectra demonstrating the photostability of PDFNPs in deionized water (left) and protein‐containing medium (right) over 3 days. (d) Fluorescence spectra of PDFNPs upon dissolution in a lipophilic solvent (THF), highlighting the distinct ratiometric peak shift from the FRET‐ON to FRET‐OFF state.

Scanning electron microscopy (SEM) confirmed the formation of monodisperse PDFNPs with diameters below 100 nm (Figure 3A). Dynamic light scattering (DLS) measurements demonstrated exceptional colloidal stability, with particle sizes remaining constant for up to 7 days in various physiological environments, including protein‐rich cell culture media and protein‐free salt solutions (Figure 3B). While previous studies often attribute such stability to the formation of a protein corona [20, 21], the inherent stability of PDFNPs in protein‐free media underscores the robust nature of our carrier‐free architecture. The optical stability and ratiometric response of PDFNPs were further evaluated under physiologically relevant conditions. The fluorescence spectra remained remarkably stable in both aqueous and protein‐rich environments for at least three days (Figure 3C). In contrast, dissolution in a lipophilic solvent triggered a dramatic spectral migration from *λ*_em_ = 580 nm (F‐ON, particulate state) to 510 nm (F‐OFF, molecular state) (Figure 3D). This distinctive robustness suggests that PDFNPs remain structurally intact, avoiding premature leakage or dissolution in protein‐rich media—mimicking the systemic circulation typical of in vivo conditions. Such stability ensures that the ratiometric signal specifically reflects the intended intracellular nanometabolism rather than non‐specific extracellular interactions.

To further evaluate their performance under practical bioimaging conditions, mono‐component nanoparticles (containing either the donor or acceptor alone) alongside the formulation‐optimized PDFNPs were subjected to confocal laser scanning microscopy. Remarkably, each nanoparticle system demonstrated exceptional resistance to photobleaching, maintaining its fluorescence integrity without distinct degradation even after 200 consecutive laser scans under standard imaging parameters (405 nm excitation, 5% laser power) (Figure S3). This robust photostability directly underscores the structural integrity of these organic matrices and confirms their superb suitability for fluorescence imaging applications.

### 2.2 Spatiotemporal Monitoring of Intracellular Nanometabolism

**FIGURE 4:**
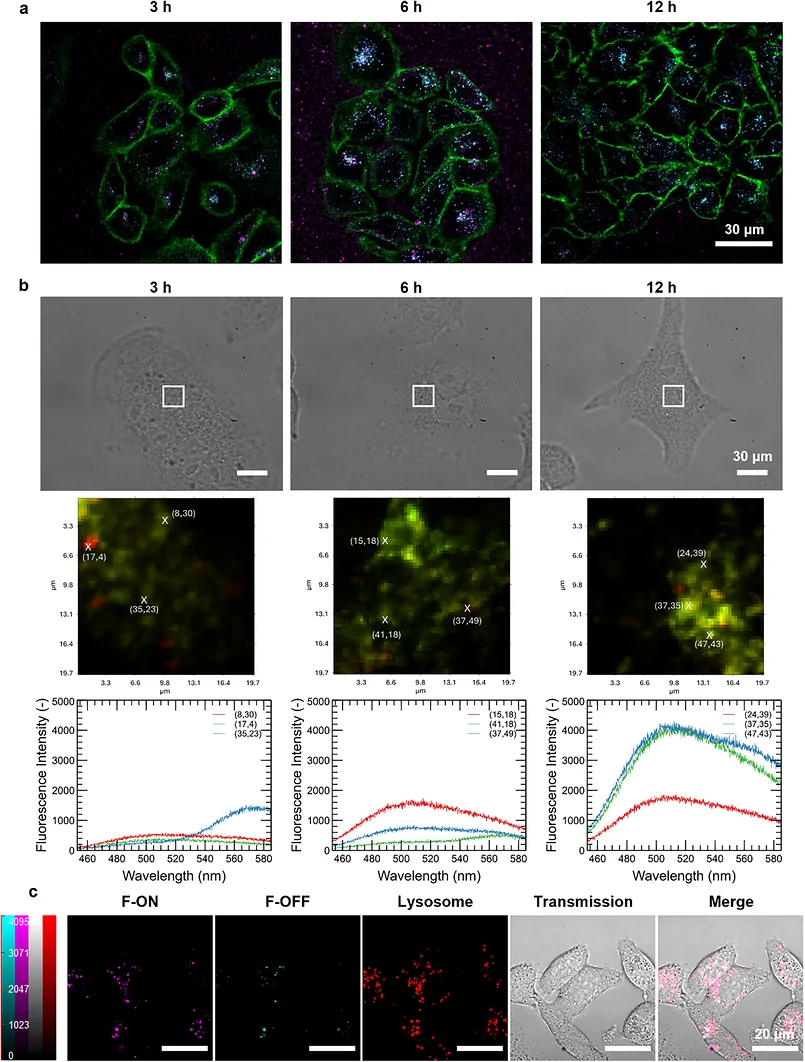
Spatiotemporal monitoring of intracellular nanometabolism and lysosomal dissolution of PDFNPs. (a) Representative CLSM images of A549 cells illustrating the time‐dependent nanometabolism of PDFNPs. The F‐ON signal (magenta) dominates at 3 h post‐internalization, followed by the emergence of the F‐OFF signature (cyan) at 6 h, and the near‐complete disassembly of nanoparticles by 12 h. Cell membranes were counterstained with CellMask Green (Scale bars: 30 µm). (b) Intracellular lambda‐scanning (spectral imaging) of A549 cells. The transmission images indicate the specific regions of interest (white squares). Point spectra acquired from individual pixels confirm a systematic shift of the emission peak, directly correlating with the PDFNP dissolution kinetics observed in vitro (Scale bars: 30 µm). (c) Colocalization analysis of PDFNPs with lysosomal trackers in A549 cells at 6 h post‐intake. The spatial overlap between the emerging F‐OFF signal and the lysosomal stain (red) confirms that the particle‐to‐molecule transition is specifically mediated within the lysosomal microenvironment (Scale bars: 20 µm).

To investigate the intracellular dynamics and metabolic fate of poorly soluble organic nanoparticles, we performed live‐cell ratiometric imaging using the A549 cell line as a primary model. Following a 2 h incubation with PDFNPs, we tracked the spatiotemporal evolution of the probes via confocal laser scanning microscopy (CLSM) at 3, 6, and 12 h post‐exposure (Figure 4A). A tri‐channel detection strategy was employed: a FRET‐mediated channel (FRET‐ON, magenta) representing the intact particulate state, a donor‐derived channel (FRET‐OFF, cyan) for the dissolved molecular state, and a membrane stain (green) for structural context.

Between 3 and 12 h, a distinct spectral transition from FRET‐ON to FRET‐OFF was observed. While PDFNPs remained structurally integrated at 3 h, a significant metabolic shift occurred at 6 h, characterized by the attenuation of the FRET‐ON signal and the concomitant emergence of the FRET‐OFF signature. By 12 h, the intracellular fluorescence had almost entirely transitioned to the FRET‐OFF state, signifying that nanoparticle disassembly and molecular dissolution had become the dominant processes. To rigorously validate these observations, we performed intracellular lambda‐scanning by scanning probe microscopy, which confirmed a clear, time‐dependent transition that encodes the molecular‐level dissolution kinetics of the nanoparticles (Figure 4B).

To pinpoint the subcellular site responsible for this transition, we conducted colocalization assays with lysosomal trackers in A549 cells at the pivotal 6 h mark. High‐resolution imaging revealed a heterogeneous distribution where both FRET‐ON and FRET‐OFF signals coexisted. Crucially, FRET‐OFF signals emerged exclusively within regions where FRET‐ON signals overlapped with lysosomes (Figure 4C). This specific spatial correlation provides definitive evidence that the lysosomal microenvironment triggers the transition from particulate assembly to the molecular state, serving as the primary engine of nanometabolism. The generality of this metabolic pathway was further investigated using the HeLa cell line. Consistently, HeLa cells exhibited a similar ratiometric shift from FRET‐ON to FRET‐OFF within the same temporal window (Figure S4A).

To validate that the dynamic, peak‐shifted ratiometric FRET response of PDFNPs is driven exclusively by intracellular nanometabolism rather than by nonspecific microenvironmental factors or intrinsic dye degradation, we synthesized and evaluated donor‐only and acceptor‐only nanoparticles as negative controls. When internalized into cells under identical experimental conditions, both the donor‐only and acceptor‐only formulations exhibited highly stable fluorescence intensities and localized intracellular signals (Figure S5A,B). Crucially, time‐course spectral tracking revealed that neither control nanoparticle exhibited any distinct peak shifts or noticeable changes in spectral profile, even after a prolonged incubation period of 12 h (Figure S5C). These results clearly demonstrate that the individual fluorophore assemblies remain photophysically intact within the intracellular environment. Therefore, this confirms that the robust spectral transition observed in the composite PDFNP platform is a unique consequence of the designed FRET disruption and membrane‐mediated molecular dissolution mechanism, thereby enabling exceptional reliability for quantitative visualization.

Crucially, this lysosome‐specific dissolution profile of PDFNPs aligns seamlessly with the metabolic fates previously documented for conventional, carrier‐free nanomedicines. This correlation emphasizes that even in the absence of an active therapeutic payload, the PDFNP platform can circumvent pharmacological interference and isolate the nanostructure's structural evolution. Consequently, this enables highly defined, unimpeded observation of nanoparticle dissolution kinetics, effectively bridging the analytical gap between surrogate characterization and actual nanomedicine behavior. Furthermore, while previous investigations were fundamentally limited by the suboptimal brightness and premature quenching of conventional fluorophores—leaving the exact locus of nanoparticle dissolution largely presumptive—the exceptional brilliance and photostability of the PDFNP platform fundamentally overcome these optical constraints. Supported by this definitive spatial colocalization (Figure 4C), this unprecedented signal‐to‐noise fidelity allows us to unambiguously establish the lysosome as the intracellular site where organic nanoparticle dissolution occurs, shifting the phenomenon from a qualitative assumption to a conclusively validated mechanism.

### 2.3 Methodological Versatility of PDFNPs in Multidimensional Heterogeneity and Tissue‐Level Tracking

To demonstrate the broad analytical utility of the PDFNP bioimaging platform, we investigated its capacity to resolve complex, interdependent nano‐bio interactions across various cellular and tissue‐level models, without being confined to a single biological environment. We first used the PDFNP framework to elucidate how structural dimensions and cellular heterogeneity jointly govern intracellular behaviors. By leveraging the fundamental principles of nucleation and growth in the reprecipitation method, larger PDFNPs were engineered by maintaining a residual concentration of organic solvent to facilitate controlled growth (see Experimental Section). This process yielded PDFNPs with diameters exceeding 400 nm, while maintaining an exceptionally high degree of spectral coincidence with their standard <100 nm counterparts (Figure S6A–C), thereby ensuring that metabolic disassembly pathways strictly dictated any subsequent optical variations.

When evaluated across different physical dimensions, the nanoparticle size dependencies of nanometabolism were distinctly mapped; the larger PDFNPs were efficiently internalized by lung‐derived A549 cells but remained predominantly in the FRET‐ON state even up to 12 h post‐exposure, whereas liver‐derived HepG2 cells revealed a discernible shift toward FRET‐OFF signatures within 6 h (Figure S6). Crucially, to further demonstrate the platform's capacity to resolve cell‐type‐specific variations, we investigated the nanometabolic kinetics of standard‐sized PDFNPs within a highly active phagocytic environment using the RAW 264.7 macrophage cell line. The resulting CLSM imaging and intracellular fluorescence spectra revealed an exceptionally rapid, near‐complete transition to the FRET‐OFF state within 3 h post‐internalization, marked by a sharp spectral red‐shift (Figure S7). Rather than merely identifying isolated cell‐line disparities, these combined findings highlight the unique capability of the PDFNP platform to sensitively and comprehensively map the heterogeneity of nanometabolism throughput—simultaneously resolving both the physicochemical dimensions (particle size) and host‐cell characteristics (epithelial cancer vs. phagocytic immune cells) that conventional stochastic imaging modalities have historically obscured. Consequently, these data solidify the PDFNP framework as a robust, highly versatile optical imaging strategy uniquely suited for dissecting multi‐dimensional nanometabolic profiles with high biological fidelity.

The analytical utility of this bioimaging platform extends beyond conventional in vitro models, providing a powerful toolkit for investigating nanoparticle dynamics within complex ex vivo tissue architectures. To validate this tissue‐level applicability, we monitored the penetration and localized processing of PDFNPs within the highly restrictive corneal epithelium. While various nanoparticles have been actively proposed as advanced vehicles to bypass the corneal barrier for enhanced ocular drug delivery [16], tracking their subsequent metabolic fate and localized dissolution kinetics within these dense, complex tissue layers have remained notoriously challenging due to the lack of high‐contrast, non‐quenched optical probes.

Our platform clearly resolved size‐dependent tissue permeability and localized transformation kinetics in real time: while the 100 nm PDFNPs successfully permeated the dense corneal barrier to a depth of approximately 4 µm within 1 h and underwent rapid dissolution, the 500 nm variants remained largely sequestered at the superficial layers (Figure S8D,E). Collectively, these distinct applications establish the PDFNP framework not as a specialized, single‐use probe but as a highly versatile and robust methodological platform capable of enabling predictable tracking across previously inaccessible biological barriers and heterogeneous cellular landscapes.

### 2.4 Mechanistic Elucidation of Lysosomal Nanometabolism

While our spatiotemporal monitoring confirmed that PDFNP dissolution occurs within lysosomes, the specific chemical or physical trigger remained to be elucidated. The lysosomal lumen is characterized by an acidic pH (4.5–5.0) and a high density of lipidic membrane structures [22]. To isolate the primary driver of the particle‐to‐molecule transition, we systematically evaluated these factors using biomimetic models.

Initially, the effect of acidity was investigated by incubating PDFNPs in aqueous buffers ranging from pH 7.0 to 3.0. Intriguingly, the fluorescence spectra remained in the FRET‐ON (magenta) state across all pH levels for over 3 days, with no detectable emergence of FRET‐OFF (cyan) emission (Figure S9A). These results demonstrate that protonation or acidity alone is insufficient to trigger the structural disassembly of the BBTP‐based matrix, suggesting that the intracellular dissolution is not a simple acid‐catalyzed response.

**FIGURE 5:**
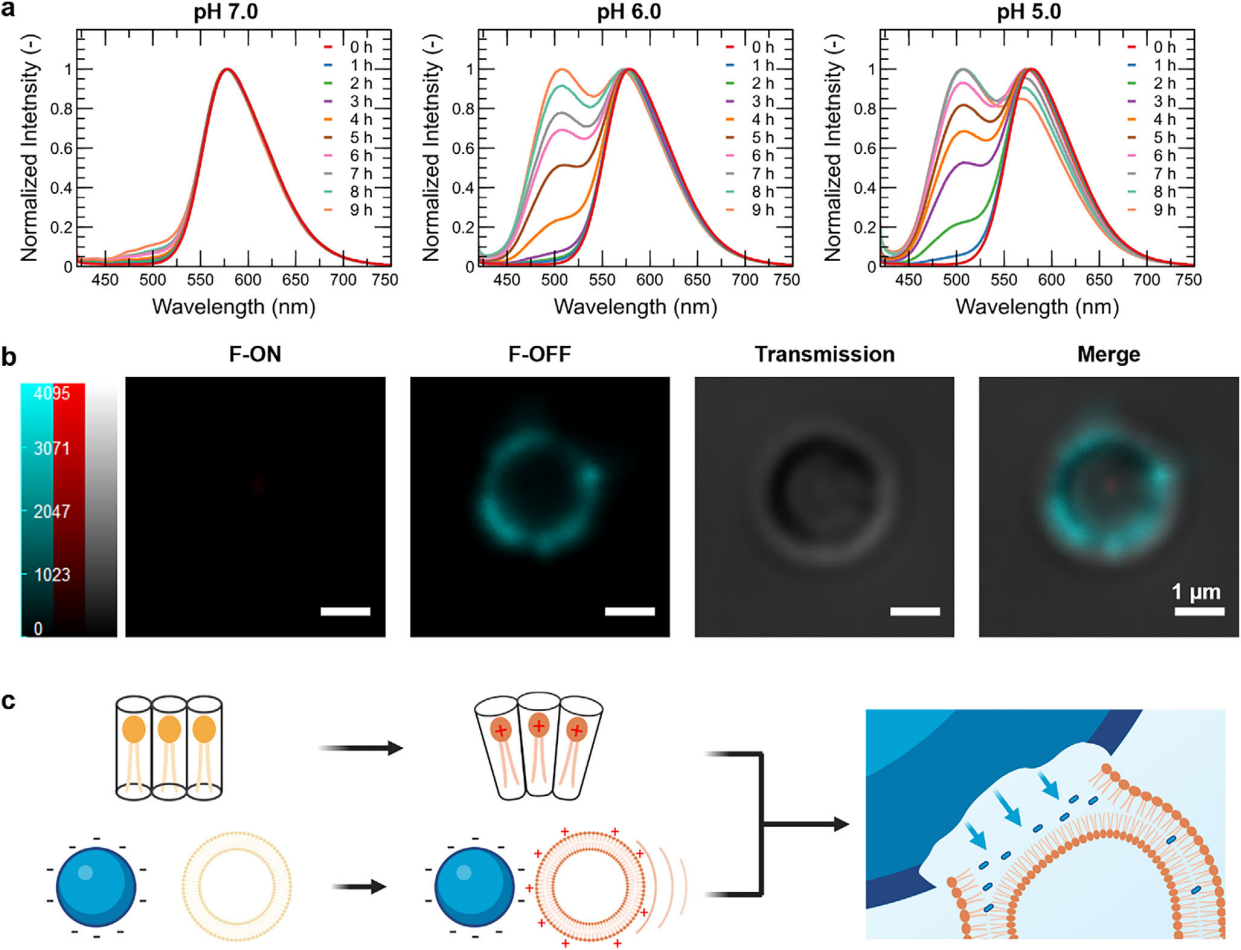
Biomimetic investigation of the synergistic, membrane‐mediated dissolution mechanism of PDFNPs. (a) Time‐resolved emission spectra of the lysosomal mimicry models, demonstrating that the ratiometric peak shift is significantly accelerated under acidic conditions (pH 5.0). (b) CLSM images capturing the localized dissolution and subsequent accumulation of BBTP molecules at the bilayer interface of GUVs at pH 4.5, confirming molecular integration into the membrane (Scale bars: 1 µm). (c) Proposed mechanism of pH‐responsive nanometabolism: Protonation of lipid headgroups induces electrostatic repulsion and membrane destabilization, exposing the hydrophobic intermembrane space. The surface properties of PDFNPs facilitate fusion with the protonated bilayer, leading to interfacial solubilization and the entrapment of dissolved molecular species within the adjacent hydrophobic domains.

Consequently, we investigated the interaction between PDFNPs and lipidic environments by constructing a lysosomal mimicry model consisting of phosphatidylcholine/cholesterol liposomes. Specifically, to capture the multifaceted microenvironment of the lysosomal lumen, the PDFNP‐liposome mixtures were systematically incubated within a series of citrate–phosphate (citric acid/disodium hydrogen phosphate) buffer systems tailored to precise pH gradients. Furthermore, continuous mechanical stirring was applied to this platform to actively replicate the high localized nanoparticle concentrations and the frequent, dynamic vesicle‐to‐particle collisions characteristic of the restricted lysosomal intra‐lumen space. While the nanoparticles remained stable in aqueous acidic buffer, the introduction of lipid bilayers triggered a discernible spectral shift from FRET‐ON (magenta) to FRET‐OFF (cyan) (Figure 5A).

To further validate that the observed dissolution arises from interaction with the intact lipid architecture rather than a nonspecific detergent‐like effect, we employed cryogenic electron microscopy (cryo‐EM). Even after 24 h of continuous stirring in the presence of PDFNPs, the cryo‐EM images clearly showed that the phospholipid bilayer structure of the vesicles remained perfectly intact (Figure S9B). This crucial finding confirms that interfacial solubilization is not the result of lipid micellization driven by degraded phospholipids, but rather a sophisticated molecular extraction process mediated by the stable bilayer interface.

Crucially, we discovered that the rate of this interfacial solubilization is highly sensitive to the surrounding pH. As the acidity increased (from pH 7.0 to 5.0), the dissolution kinetics of PDFNPs in the presence of liposomes were significantly accelerated. This suggests that the acidic environment of the lysosome does not act as a direct catalyst for the organic matrix itself but rather functions synergistically with the lipid bilayer. We propose that acidic pH induces localized protonation of phospholipid head groups, thereby decreasing bilayer packing density and increasing solvent capacity. This destabilized interface facilitates the more efficient extraction of hydrophobic BBTP molecules from the nanoparticle core. In this synergistic model, the lysosomal membrane acts as an acid‐activated, 2D solvent that thermodynamically and kinetically favors the molecularly dissolved state, providing a comprehensive explanation for the rapid phase transition observed specifically within the acidic, lipid‐rich lysosomal interior. While lipid interactions are the primary driver, dissolution kinetics were significantly accelerated under acidic conditions compared to neutral pH. This suggests a synergistic mechanism in which the acidic environment may destabilize the nanoparticle surface or enhance molecular partitioning. To provide direct visual evidence, we employed Giant Unilamellar Vesicles (GUVs) as a high‐resolution biomimetic model (Figure 5B).

PDFNPs were integrated during the GUV fabrication process under acidic conditions to simulate lysosomal sequestration. CLSM image of the resulting GUVs revealed a dominant FRET‐OFF (cyan) signal uniformly distributed across the spherical lipid boundaries (Figure 5B). This uniform membrane integration confirms that the BBTP molecules were not merely adsorbed but were successfully internalized into the hydrophobic core of the lipid bilayer, validating the efficiency of membrane‐mediated interfacial solubilization.

Complementing these structural observations, cryogenic correlative light and electron microscopy (cryo‐CLEM) provided high‐resolution, multi‐modal evidence of the nanometabolic process. By correlating fluorescence signals with ultrastructural changes, cryo‐CLEM successfully visualized the morphological transition from compact, solid‐state PDFNPs to partially disassembled, loose aggregates (Figure S9D). This direct visualization of the physical breakdown of the nanoparticles at the nanoscale perfectly validates our ratiometric optical observations, providing definitive proof of the structural disassembly that precedes molecular integration into the lysosomal mimicry membrane.

Based on the cumulative evidence from intracellular imaging and biomimetic GUV studies, we propose a novel membrane‐triggered dissolution mechanism that shifts the focus from the nanoparticle's intrinsic sensitivity to the dynamic biophysical response of the lysosomal interface (Figure 5C). In this model, the acidic lysosomal lumen induces protonation of phospholipid head groups in the lysosomal membrane, generating localized electrostatic repulsion that leads to lateral expansion of the lipid bilayer. This transition increases the membrane's free volume and creates transient hydrophobic gaps, transforming the bilayer into an energetically favorable, 2D solvent.

Consequently, the lipophilic BBTP molecules are efficiently extracted from the solid nanoparticle core and partitioned into the destabilized membrane interface. This interfacial solubilization facilitates the rapid transition from the FRET‐ON particulate state to the FRET‐OFF molecular state, thereby leveraging the organelle's natural physiological evolution as a precise trigger for nanometabolic activation.

Simultaneously, the protonation converts the lipid bilayer surface into a positively charged interface, which undergoes robust electrostatic attraction with the negatively charged nanoparticle surface—a charge state driven by the selective adsorption of hydroxide ions onto its hydrophobic domains. This charge complementarity actively accelerates closer surface‐to‐surface interactions, drawing the nanoparticle into tight contact with the membrane. Consequently, the lipophilic BBTP molecules are efficiently extracted from the solid nanoparticle core and partitioned into the destabilized membrane interface. This interfacial solubilization facilitates the rapid transition from the FRET‐ON particulate state to the FRET‐OFF molecular state, effectively utilizing the natural physiological evolution of the organelle as a precise trigger for nanometabolic activation.

**FIGURE 6:**
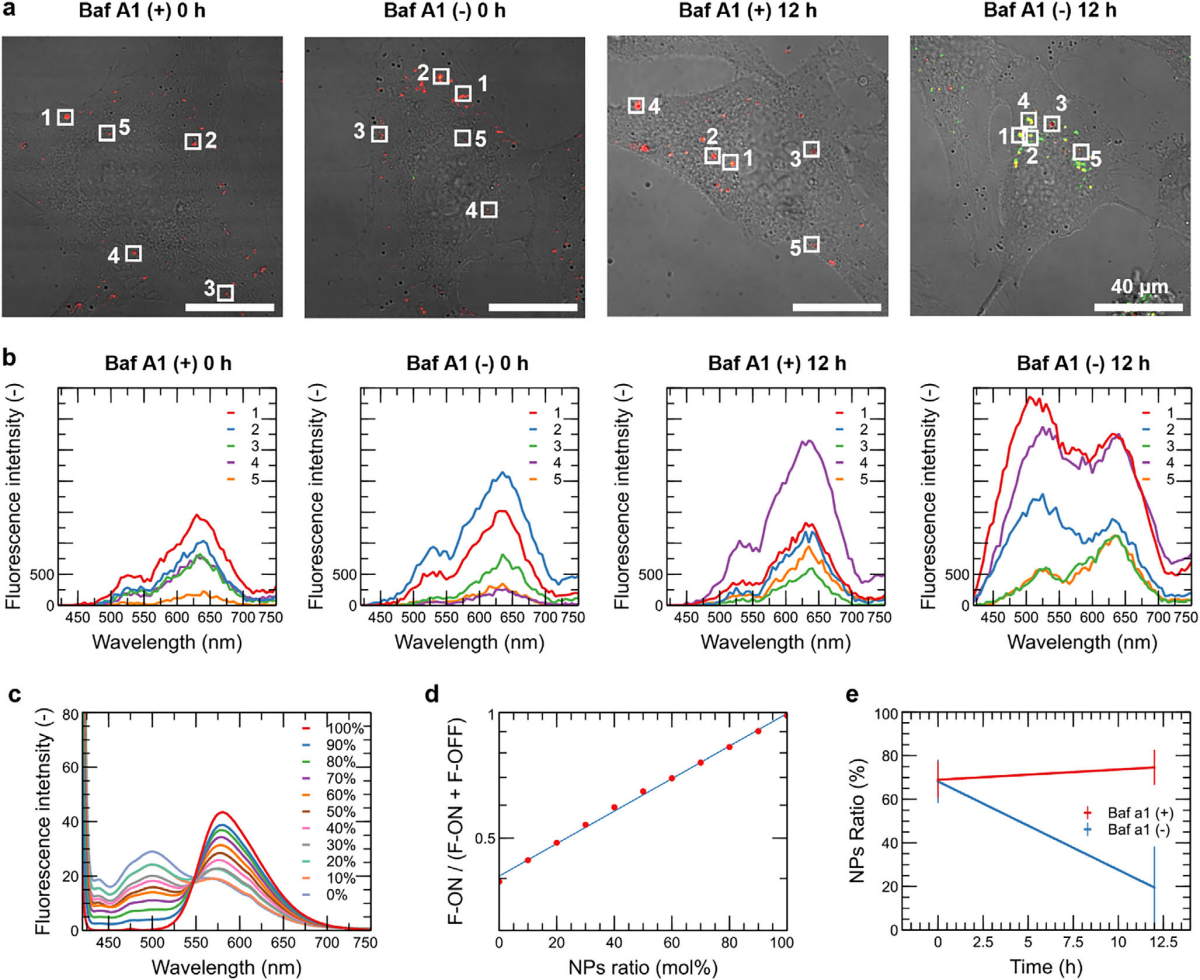
In vitro validation of the membrane‐triggered dissolution mechanism and quantitative nanometabolic profiling via lysosomal neutralization. (a) CLSM images of HeLa cells incubated with PDFNPs for 0 and 12 h in the absence (control) or presence of a lysosomal acidification inhibitor (Bafilomycin A1‐treated) (Scale bars: 40 µm). (b) Intracellular fluorescence spectra acquired from the corresponding areas in (a), revealing the time‐dependent spectral migration from FRET‐ON to FRET‐OFF states. (c) Fluorescence emission spectra of the biomimetic calibration model, acquired by systematically mixing PDFNPs and fully dissolved BBTP‐loaded liposomes across a defined gradient of nanoparticle fractions (from 0% to 100%). (d) Standard calibration curve fitted by plotting the remaining intact nanoparticle (NP) ratio against the normalized ratiometric fluorescence intensity, mathematically defined as $\frac{I_{FRET-ON}}{I_{FRET-ON}+I_{FRET-OFF}}$, derived from the spectra in (c). *R* ^2^ =  0.998 (e) Quantitative nanometabolic kinetics demonstrating the intracellular NP ratio as a function of incubation time 0 h (*p* > 0.1) to 12 h (*p* < 0.01, *p* values were calculated using student's t‐test) for both the control and Bafilomycin A1‐treated groups, calculated by the cell‐derived spectra from (b) into the calibration framework established in (d) (5 spots, *n*  =  5). Standard deviation was shown by error bars. Statistical analysis: Data are presented as mean ± SD (*n*  =  5 independent intracellular regions of interest per group). Statistical significance was assessed using a Student's two‐sided independent t‐test comparing the control and Bafilomycin A1‐treated groups at each time point (exact *p* values are indicated: *P*  =  0.4307 at 0 h, *P*  =  0.0024 at 12 h).

To investigate whether this hypothesized mechanism accurately governs the nanoparticle fate within living cells, we monitored the intracellular disassembly of PDFNPs in the presence of a lysosomal acidification inhibitor. Bafilomycin A1 was employed to selectively inhibit the vacuolar H+‐ATPase, thereby preventing the acidification of the lysosomal lumen. Following the co‐incubation of PDFNPs with Bafilomycin A1 in HeLa cells, the intracellular fluorescence signatures were comprehensively analyzed at the 12 h post‐exposure mark (Figure 6A,B). Remarkably, while the untreated control group exhibited a distinct and clear spectral migration toward the FRET‐OFF state within 12 h, the Bafilomycin A1‐treated cells retained the majority of their fluorescence signals in the FRET‐ON state.

To translate these fluorescence variations into a rigorous quantitative metric, the intracellular spectra were decoded using a biomimetic liposome calibration model. Liposomes co‐incorporating the BBTP donor and acceptor dyes at the identical stoichiometric ratio as the PDFNPs were engineered to simulate the fully dissolved molecular state within a lipid bilayer matrix. By systematically mixing these BBTP‐loaded liposomes with pristine PDFNPs at designated ratios, we acquired a series of standard fluorescence spectra (Figure 6C). These profiles were subsequently utilized to construct a robust calibration curve that mathematically correlates the specific fluorescence peak separation with the exact percentage of intact nanoparticles remaining in the system (Figure 6D).

By feeding the experimentally derived intracellular spectra into this calibration curve, we achieved a highly precise, quantitative comparison of the nanoparticle‐to‐molecule transition kinetics (Figure 6E). The quantitative readout conclusively verified that lysosomal neutralization via Bafilomycin A1 markedly suppressed the molecular dissolution rate of PDFNPs inside the cells. This robust alignment between the cell‐free model and in vitro imaging provides definitive validation for our proposed membrane‐triggered nanometabolic mechanism. Furthermore, this quantification directly highlights the methodological versatility of the PDFNP platform for advanced nanomedicine screening.

## 3 Conclusion

Fluorescence‐based tracking of nanoscale materials within the complex intracellular environment is frequently compromised by environmental quenching and the suboptimal brilliance of conventional fluorophores, including those employed in standard FRET architectures [23, 24]. PDFNPs circumvent these intrinsic bottlenecks by integrating exceptionally bright, photostable donor‐acceptor pairs that yield unambiguous ratiometric spectral signatures, enabling definitive discrimination between intact nanoparticles and their dissolved molecular counterparts. Time‐resolved confocal imaging successfully captured the stepwise kinetics of lysosomal degradation over a 6–12 h window. These data provide rigorous quantitative insights into nanoparticle fate as a function of both cell lineage and particulate dimensions—parameters that were previously obscured when using stochastic ON/OFF‐type FRET systems.

While the efficacy and cellular internalization of carrier‐free nanomedicines have been extensively documented, their subsequent intracellular disassembly profiles—and the precise mechanisms driving these metabolic transformations—have remained largely obscured [25, 26, 27]. Utilizing the PDFNP platform, we successfully resolved these long‐standing questions by establishing a clear spatiotemporal profile of lysosomal processing. Crucially, while previous understandings of carrier‐free nanoparticle fate remained purely speculative, our systematic evaluation directly demonstrates that the acidic microenvironment and synergistic lipid‐bilayer interactions are the definitive governing factors driving nanometabolism. By elucidating this membrane‐mediated disassembly pathway, this work offers a validated mechanistic model that fills a critical knowledge gap in nanomedicine, reinforcing the fundamental design principles required to predict and govern therapeutic bioavailability.

Beyond providing mechanistic clarity, PDFNPs serve as high‐fidelity surrogates for the assembly of poorly soluble drug molecules. While the introduction of a third therapeutic component often risks destabilizing host nanostructures, the exceptional molecular affinity within the BBTP matrix accommodates hydrophobic drug payloads without compromising the core FRET ratiometric integrity. Building upon this structural robustness, further in vivo pharmacokinetic tracking and therapeutic evaluation utilizing drug‐loaded PDFNPs are actively underway, paving the way for comprehensive theragnostic applications in future studies. The ability to systematically probe cell‐type‐ and size‐dependent dissolution kinetics addresses a long‐standing critical gap in nanomedicine. Furthermore, the modular tunability of PDFNP emission properties via alternative donor–acceptor combinations within the BBTP derivative library underscores the formulation versatility of these peak‐shifted nanoprobes for future molecular engineering of carrier‐free organic matrices. Collectively, this strategy establishes a quantitative foundation for analyzing nanoparticle transformation and in vivo behavior, offering a transformative toolkit for the rational development of next‐generation theragnostic systems.

## 4 Experimental Section

### 4.1 Preparation and Characterization of PDFNPs

PDFNPs were prepared by the reprecipitation method. MES‐BBTP and TPA‐BBTP were dissolved in THF and adjusted to a concentration of 4 mM to prepare PDFNPs containing both FRET donors and acceptors. Subsequently, 100 µL of the THF solution containing each component was rapidly injected into 1.9 mL of deionized water being stirred at 1300 rpm. PDFNPs and THF solutions containing the donor and acceptor at a 90:10 ratio were applied. The prepared PDFNPs dispersion was stored at 4°C in the dark without further modification. Under the same conditions, the dispersion was diluted with deionized water or physiological medium (DMEM containing 10% FBS or Hank's balanced salt solution). The particle size distribution was measured by DLS (Malvern Zetasizer NanoZS) immediately after dilution at 1, 2, 3, and 7 days. The fluorescence spectra were measured after dilution at 1,2, and 3 days. Fluorescence spectra were measured by a fluorescence spectrometer (Hitachi F‐7000) after diluting the PDFNPs dispersion with a deionized water‐THF mixed solvent of varying ratios. PDFNPs dispersion was deposited on a polycarbonate membrane with 50 nm pores, filtered, and observed utilizing FE‐SEM (Hitachi S‐4800). Additionally, a THF solution adjusted to the same composition and concentration as the PDFNPs dispersion was measured using a UV/Vis/NIR spectrometer (JASCO V‐570) and a fluorescence spectrometer to determine the absorption and fluorescence spectra. Fluorescence lifetime measurements were performed on PDFNPs as well as nanoparticles composed solely of the donor or acceptor, and their corresponding THF solutions. The THF solution containing the same composition as PDFNPs was employed for quantum yield measurement. To evaluate the amorphous structural states of the nanoparticles, powder X‐ray diffraction (PXRD) patterns were recorded on a Rigaku SmartLab3G diffractometer. The instrument was operated using Cu K beta radiation as the X‐ray source at an accelerating voltage of 40 kV and a tube current of 30 mA. Before analysis, the dispersions of PDFNPs, MES‐BBTP NPs, and TPA‐BBTP NPs were dried to obtain fine solid powders. PXRD data were collected over a 2Theta/Theta scanning with a step size of 0.0 at room temperature. To evaluate the photostability of the organic nanoprobes under continuous photo‐irradiation, confocal laser scanning microscopy (CLSM) imaging was performed using an Olympus FV1000 microscope. For sample preparation, 1 mL of each nanoparticle dispersion (PDFNPs, TPA‐BBTP NPs, and MES‐BBTP NPs) was deposited onto a glass‐bottom dish and gently dried under a continuous argon gas stream. The dried samples were visualized using a 10 times objective lens. Continuous photo‐irradiation was executed by performing 200 consecutive laser scans across the identical region of interest under standard imaging parameters. The excitation wavelength was set to 405 nm, with a transmission power of 5.0%. The fluorescence emission signals were simultaneously monitored and captured through two designated detection channels—Channel 1 (Ch1: 460 – 520 nm for green emission) and Channel 2 (Ch2: 650 – 750 nm for red emission)—to track potential photobleaching or degradation of the nanostructured matrices.

### 4.2 Intracellular Imaging

HeLa cells were cultured in DMEM supplemented with 10% FBS, 1% penicillin‐streptomycin, and 1% GlutaMax at 37°C and 5% CO_2_. Subsequently, HeLa cells (2 × 10^5^ cells per dish) were seeded into the glass‐bottom dish and cultured overnight at 37°C and 5% CO_2_. After overnight incubation, the HeLa cells were exposed to a PDFNP dispersion (10‐fold diluted in DMEM) for 2 h. For the lysosomal neutralization experiments, 200 nM Bafilomycin A1 was administered for 6 h prior to a 2 h PDFNP exposure period. After the incubation, the cells were thoroughly washed with fresh culture medium to remove any non‐internalized extracellular nanoparticles. After 2 h of PDFNPs exposure, HeLa cells were washed with culture medium. CLSM imaging and lambda scan were performed using a confocal fluorescence microscope (Olympus FV1000) at 0, 6, 12, and 24 h after PDFNPs exclusion. After 24 h of incubation, lysosomal staining was performed with LysoPrime Deep Red for 30 min, followed by CLSM imaging. PDFNP was excited with the 405 nm emission line of a diode laser, and LysoPrime Deep Red was excited with the 635 nm emission line of a helium‐neon laser. F‐ON, F‐OFF, and LysoPrime Deep Red signals were measured sequentially using the 650–750, 425–475, and 650–750 nm emission ranges, respectively.

A549 cells (2 × 10^5^ cells per dish) were seeded in the glass‐bottom dish and cultured overnight at 37°C and 5% CO_2_. After overnight incubation, the A549 cells were exposed to a PDFNP dispersion (10‐fold diluted in DMEM) for 2 h. After 2 h of PDFNPs exposure, A549 cells were washed with culture medium. CLSM imaging was performed using a confocal fluorescence microscope (Nikon A1 H25) at 3, 6, and 12 h after PDFNPs exclusion. Cell membranes were stained with CellBrite for 10 min prior to CLSM imaging, then washed with DMEM to complete sampling. PDFNP was excited with the 402.7 nm emission line of a diode laser, and CellBrite was excited with the 637.8 nm emission line of a helium‐neon laser. F‐ON and F‐OFF signals were measured using the 662.5–737.5 and 435–485 nm emission bands, respectively. CellBrite was measured using the 662.5–737.5 nm emission range.

To avoid laser‐induced phototoxicity and potential oxidative cellular damage caused by prolonged exposure to the short‐wavelength excitation laser, a time‐course imaging approach using independent cell samples was adopted. At each specified time point, multiple randomized fields of view were captured, ensuring that each measured cell group was exposed to the excitation laser only once.

Scanning probe microscopy observations were performed on cells treated with PDFNPs under the same culture conditions. PDFNPs were excited with the 405 nm emission line of a diode laser. Lysosome staining was performed with LysoPrime Deep Red for 30 min after 6‐h incubation following PDFNP exclusion, followed by CLSM imaging.

Larger‐sized PDFNPs were fabricated for comparative analysis. MES‐BBTP and TPA‐BBTP were dissolved in THF and adjusted to a concentration of 4 mM to prepare PDFNPs containing both FRET donors and acceptors. Subsequently, 100 µL of the THF solution containing MES‐BBTP and TPA‐BBTP at a 9:1 ratio was rapidly injected into 1.9 mL of a mixed solvent of deionized water and THF (8:3, v/v) being stirred at 1500 rpm. Then the stirring was stopped for 2 min to allow particle growth, followed by the immediate injection of 1.8 mL of PDFNP dispersion into 0.9 mL of deionized water to halt further growth. Residual organic solvent in the fabricated PDFNPs dispersion was removed using a rotary evaporator, and the concentration was adjusted by adding deionized water. The final concentration of PDFNPs dispersion was 0.2 mM, and the dispersion was subsequently administered to A549 and HepG2 cells for CLSM imaging.

A549 or HepG2 cells (2 × 10^5^ cells per dish) were seeded into the glass‐bottom dish and cultured overnight at 37°C and 5% CO_2_. After overnight incubation, the cells were exposed to a PDFNP dispersion (10‐fold diluted in DMEM) for 2 h. After 2 h of PDFNPs exposure, cells were washed with culture medium. CLSM imaging was performed 6 and 12 h after PDFNPs exclusion.

RAW264.7 cells (2 × 10^5^ cells per dish) were cultured in DMEM supplemented with 10% FBS, seeded in the glass bottom dish, and cultured overnight at 37°C, 5% CO2. After overnight incubation, the cells were exposed to a PDFNP dispersion (10‐fold diluted in DMEM) for 2 h. After 2 h of PDFNPs exposure, cells were washed with culture medium. CLSM imaging was performed 3 and 6 h after PDFNPs exclusion.

### 4.3 Lysosomal Mimicry Assays

The lysosomal mimicry model consisted of liposomes, acidic buffer, and PDFNPs. The liposomes were prepared by an extrusion method. DPPC and cholesterol were mixed in a 7:3 ratio to mimic a biological membrane. The lipid mixture was dissolved in chloroform, and then chloroform was removed by a rotary evaporator to form a dry lipid film on the bottom of a glass flask. The dry lipid film was further dried overnight under vacuum to remove residual solvent. The dry lipid film was hydrated at 55°C for 1 h with deionized water. Hydration was performed to achieve a total lipid concentration of 1 mg/mL. The resulting lipid membrane dispersion was extruded 11 times through a polycarbonate membrane with 400 nm pores.

The prepared liposome dispersion, PDFNPs dispersion, and diluted McIlvaine buffer were mixed at a volume ratio of 6:3:1 to construct a lysosomal mimicry model. The model was stirred at 800 rpm to induce interactions between PDFNPs and liposomes. Fluorescence spectra were measured by a fluorescence spectrometer at 1‐h intervals for 9 h after the model was formed.

Giant unilamellar vesicles were prepared by a gentle hydration method. First, DPPC and cholesterol were dissolved in chloroform at a molar ratio of 7:3, and the final total lipid concentration was adjusted to 2 mM. Then, 20 µL of the lipid solution was deposited on the bottom of a microtube. Chloroform was evaporated using a gentle argon gas stream, and the remaining solvent was removed under reduced pressure overnight. Subsequently, hydration was performed at 55°C for 24 h using a 7:3 mixture of PDFNP dispersion and diluted McIlvaine buffer at pH 4.5. CLSM imaging was performed after hydration. PDFNP was excited with the 405 nm emission line of a diode laser. F‐ON and F‐OFF signals were measured sequentially using the 600–700 and 420–500 nm emission ranges, respectively.

### 4.4 Optimization of PDFNPs

PDFNPs of various donor‐to‐acceptor ratios were prepared by the reprecipitation method. MES‐BBTP and TPA‐BBTP were dissolved in THF and adjusted to a concentration of 4 mM to prepare PDFNPs containing both FRET donors and acceptors. Subsequently, 100 µL of the THF solution containing the donor and acceptor at ratios of 99:1, 90:10, 70:30, 50:50, and 30:70 was rapidly injected into 1.9 mL of deionized water being stirred at 1300 rpm. The dispersion of PDFNPs and THF solutions prepared at each donor‐to‐acceptor ratio was analyzed using a fluorescence spectrometer.

### 4.5 Calibration of the Separation Ratio of PDFNPs

To calibrate the quantitative relationship between the molar fractions of the dissolved FRET donors/acceptors and their corresponding ratiometric fluorescence spectral shifts, lipid bilayer vesicles co‐incorporating the BBTP chromophores were prepared. A dry lipid film was formed by dissolving dipalmitoylphosphatidylcholine (DPPC) in chloroform alongside a precise mixture of TPA‐BBTP and MES‐BBTP dyes at a 9:1 stoichiometric ratio—strictly replicating the composition of the PDFNPs—followed by solvent evaporation using a rotary evaporator. The resulting lipid film was kept under a deep vacuum overnight to ensure the complete removal of any residual organic solvent. The dried film was subsequently hydrated with deionized water at 55°C to yield a final concentration of 5 mg/mL DPPC, 0.018 mM TPA‐BBTP, and 0.002 mM MES‐BBTP. Following the hydration process, the dispersion was extruded 11 times through a polycarbonate membrane with a 400 nm pore size.

For the standard calibration matrix, the engineered BBTP‐loaded liposome dispersion and the pristine PDFNP dispersion were both diluted to an equivalent total chromophore concentration of 0.02 mM and systematically blended at designated volumetric fractions (ranging from 0% to 100% nanoparticle ratios). Fluorescence emission spectra were then recorded for each mixed suspension. To mathematically decode the dissolution kinetics from the acquired spectra, the fraction of the FRET‐ON particulate state was calculated based on the ratiometric emission intensities extracted from the prominent FRET‐ON (λem = 580 nm) and FRET‐OFF (λem = 510 nm) coordinate peaks.

### 4.6 Cell Viability Assay

A549 cells were cultured in DMEM supplemented with 10% FBS, 1% penicillin‐streptomycin, 1% GlutaMax, and 0.1% Geneticin at 37°C and 5% CO_2_. HMEC cells were cultured in MEGM Bulletkit at 37°C and 5% CO_2_. HepG2 cells were cultured in DMEM supplemented with 10% FBS and 1% penicillin‐streptomycin at 37°C and 5% CO_2_. HCT‐116 cells were cultured in DMEM supplemented with 10% FBS at 37°C and 5% CO2. A549, HeLa, HMEC, and HepG2 cells were seeded in 96‐well plates at a density of 2 × 10^5^ cells/mL. After 24 h, culture medium was refreshed, and PDFNPs were added to cells. After 48 h of incubation, cells were washed with culture medium and stained with Cell Counting Kit‐8 for 1 h. After staining, the solution was measured for absorbance using a microplate reader. The measured absorbance values were used to calculate cell viability.

### 4.7 PDFNPs Structural Integrity Assay

PDFNPs dispersion and diluted McIlvaine buffer were mixed at a volume ratio of 1:9 to evaluate the structural integrity of PDFNPs under acidic conditions. Bovine serum albumin was added at a concentration of 1 mg/mL to prevent aggregation of PDFNPs. The system was stirred at 500 rpm, and the fluorescence spectrum was measured by a fluorescence spectrometer at 1‐day intervals for 3 days after the system was formed.

### 4.8 In Vivo Imaging

The Ethical Committee of the Graduate School of Medicine, Tohoku University, approved the protocol as #2022MdA‐015. The methods were carried out in accordance with the approved guidelines.

PDFNP dispersion was administered to the rat cornea for in vivo imaging. Sprague‐Dawley rats (6‐week‐old male) were obtained from Japan SLC, Inc. MES‐BBTP and TPA‐BBTP were dissolved in THF and adjusted to 8 mM to prepare PDFNPs containing both FRET donors and acceptors. Polysorbate‐80 was added to the THF solution at a concentration of 1 mg/mL as a surfactant to achieve a higher concentration of PDFNPs dispersion. Subsequently, 200 µL of BBTP solution at a 9:1 ratio was rapidly injected into 2.0 mL of deionized water being stirred at 1500 rpm. Additionally, larger‐sized PDFNPs were prepared. 200 µL of BBTP solution at a 9:1 ratio was rapidly injected into 1.8 mL of a mixed solvent of deionized water and THF (8:3, v/v) being stirred at 1500 rpm. Then the stirring was stopped for 20 s to allow particle growth, followed by the immediate injection of 1.8 mL of the PDFNP dispersion into 0.9 mL of deionized water to halt further growth. Residual organic solvent in both fabricated PDFNP dispersions was removed using a rotary evaporator, and the concentration was adjusted by adding deionized water. The dispersion was further adjusted to contain 0.9% NaCl by dilution with 9% NaCl aqueous solution. The final concentration of the PDFNPs dispersion was 0.76 mM, and the PDFNPs dispersion was subsequently administered to the rat eye. 1 h after administration, the cornea was rinsed with saline. Following washing, the rat was sacrificed, and the cornea was excised for CLSM imaging. PDFNP was excited with the 405 nm emission line of a diode laser. F‐ON and F‐OFF signals were measured sequentially using the 600–700 and 410–500 nm emission ranges, respectively.

### 4.9 Cryo‐EM and Cryo‐CLEM Imaging

The lysosomal mimicry model, immediately after preparation or after 24 h of stirring at 500 rpm, and the PDFNPs dispersion were subjected to cryogenic electron microscopy. Bovine serum albumin was added to the PDFNP dispersion to prevent adsorption of PDFNPs onto hydrophobic copper grids. Copper grids with 200‐mesh, covered with a holey carbon film (Quantifoil R2/2), were hydrophilized in advance using a glow‐discharge device (JEOL JEC‐3000FC). A 3 µL aliquot of PDFNPs ‐containing sample solutions was applied to each grid. The grids were then blotted with filter paper under 95% humidity and at 25°C and rapidly vitrified by plunging into liquid ethane using a semiautomatic EM GP2 plunger (Leica Microsystems). The prepared grids were stored in liquid nitrogen until cryo‐EM and cryo‐CLEM analysis. Cryo‐EM observations were performed using a CRYO ARM 300 II microscope (JEOL Ltd., Japan) operated at an acceleration voltage of 300 kV. Images were acquired at a nominal magnification of 12 000 for 0 h and 15 000 for 24 h. Cryogenic light microscopy was carried out using a THUNDER Cryo CLEM system (Leica Microsystems GmbH, Germany). The F‐OFF signal was excited in the 327–383 nm emission range and measured sequentially in the 435–485 nm emission range. The acceptor signal was excited in the 450–490 nm emission range and measured sequentially in the 500–550 nm emission range.

### 4.10 Statistical Analysis

All quantitative biological and spectroscopic data are presented as mean ± standard deviation (SD) derived from at least three independent experiments unless otherwise specified. For intracellular dissolution kinetics, sample sizes (*n*  =  5) represent distinct, independent intracellular regions of interest (ROIs). Prior to statistical analysis, data were evaluated for normality and homogeneity of variance; no data points or outliers were excluded. Statistical significance between experimental groups (control vs. Bafilomycin A1‐treated groups) was determined using a Student's two‐sided independent t‐test. All statistical tests were performed at a significance level of α  =  0.05, where *P* < 0.05 was considered statistically significant. Exact *p* values are provided directly in the respective figure legends to indicate statistical significance. All statistical calculations, descriptive statistics (mean and SD), two‐sided t‐tests, and data processing were performed using Microsoft Excel (Microsoft 365).

## Author Contributions

**Jinwoo Sung**: conceptualization, investigation, validation, methodology, Writing – original draft, Writing – review and editing, visualization. **Yutaro Miki**: investigation, methodology. **Farsai Taemaitree**: methodology, investigation, validation, visualization, data curation, conceptualization. **Ryuju Suzuki**: writing – review and editing. **Toru Nakazawa**: methodology, validation. **Satoshi Katsube**: methodology, visualization, investigation. **Keita Tanita**: writing – review and editing. **Keisuke Kawakami**: methodology, investigation, visualization. **Daisuke Unabara**: investigation, methodology, visualization. **Tasuku Hamaguchi**: methodology, investigation, visualization. **Minsang Kim**: investigation, visualization, methodology. **Akihide Hibara**: methodology, validation. **Kota Sato**: investigation, methodology. **Koji Yonekura**: funding acquisition, methodology, validation, resources. **Atsushi Wakamiya**: methodology, validation, resources. **Hitoshi Kasai**: funding acquisition, writing – review and editing, supervision, resources, project administration, conceptualization. **Hiroshi Uji‐i**: supervision, resources, methodology, validation, funding acquisition. **Yoshitaka Koseki**: funding acquisition. **Kunikazu Ishii**: investigation, visualization, methodology.

## Conflicts of Interest

The authors declare no conflicts of interest.

## Supporting information




**Supporting File**: advs77509‐sup‐0001‐SuppMat.docx.

## Data Availability

The data that supports the findings of this study are available in the supplementary material of this article.
