# Supplementary material for: Quantitative Visualization of Intracellular Nanometabolism Using Peak‐Shifted Dual‐State Emissive FRET Nanoprobes
Source: Adv Sci (Weinh). 2026 Sep 6:e77509. Online ahead of print. doi: 10.1002/advs.77509 (PMC13547703; doi:10.1002/advs.77509)
Supplement: Supplementary file 1 [file ADVS-9999-e77509-s001.docx]

Supporting Information

**Figure S1.** Optimization of the donor-to-acceptor ratio for maximized ratiometric contrast. Fluorescence emission spectra of PDFNPs fabricated at varying MES-BBTP (donor) to TPA-BBTP (acceptor) molar ratios: 99:1, 90:10, 70:30, 50:50, and 30:70. Each panel compares the emission profiles of the nanoprobes in their assembled particulate state (red lines) and molecularly dissolved state (blue lines). The 90:10 ratio was identified as the optimal composition, providing the highest sensitivity and most pronounced spectral shift for monitoring dissolution dynamics.

**Figure S2.** Control experiments with single-component nanoprobes and quantitative calibration of the FRET response. a) DLS profiles illustrating the colloidal stability of individual MES-BBTP and TPA-BBTP nanoparticle dispersions in water over 7 days. b,c) Absorption and fluorescence emission spectra of MES-BBTP and TPA-BBTP in their nanoparticulate (water, red lines) and dissolved (THF, blue lines) states. d) Fluorescence emission spectra (λ_ex=405nm) of PDFNPs (red line) and a physical mixture of separate single-component nanoprobes (MES-BBTP and TPA-BBTP nanoparticles mixed at a 1:9 stoichiometric ratio, blue line). The total concentration of the BBTP molecule in both groups was adjusted to 2 μM to confirm that the ratiometric signature is governed exclusively by intra-particle molecular co-packing rather than by non-specific inter-particle collisions. e) Powder X-ray diffraction (PXRD) patterns of PDFNPs (green line), mono-component MES-BBTP nanoparticles (red line), and TPA-BBTP nanoparticles (blue line). All three samples exhibit a characteristic broad halo profile with a prominent void pattern and no detectable crystalline Bragg reflections, confirming that the nanoparticulation process yields entirely amorphous molecular assemblies across all formulations.

**Figure S3.** Time-resolved photostability evaluation of the engineered nanoprobes via continuous confocal laser scanning. Confocal image series of formulation-optimized PDFNPs, mono-component TPA-BBTP NPs, and MES-BBTP NPs deposited on glass substrates, monitored as a function of laser scan repetitions (up to 200 cycles). The sample matrices were continuously excited via a 405 nm laser beam (5.0% transmittance) under a x10 oil-immersion objective lens. The preservation of fluorescence intensity was tracked simultaneously through Ch1 (460-520 nm, green emission) and Ch2 (650-750 nm, red emission), confirming the exceptional photobleaching resistance of the molecular frameworks under demanding imaging parameters. Scale bars: 300 μm.

**Figure S4.** Generality of intracellular nanometabolism and long-term lysosomal sequestration in HeLa cells. a) Representative CLSM images of HeLa cells demonstrating the time-course nanometabolism of PDFNPs. The F-ON signal (magenta) is dominant immediately after the 2 h incubation (0 h post-wash), followed by the emergence of the F-OFF signal (cyan) at 6 h and near-complete dissolution by 12 h. Scale bars: 10 μm. White squares indicate regions selected for spectral interrogation. b) Intracellular fluorescence spectra acquired from the corresponding areas in (a), revealing a time-dependent emission peak shift that matches the PDFNP dissolution profile. c) Long-term colocalization analysis of PDFNPs with lysosomal trackers in HeLa cells at 24 h post-internalization. The persistent spatial overlap between the dominant F-OFF signals and lysosomal dye (red) confirms the entrapment of dissolved molecular species within the lysosomal microenvironment. Scale bars: 20 μm.

**Figure S5.** Intracellular stability and spectral tracking of control nanoparticles. a,b) Confocal laser scanning microscopy (CLSM) images of HeLa cells incubated with acceptor-only nanoparticles (top, a) and donor-only nanoparticles (bottom, b) for 3 h, 6 h, and 12 h under identical biological conditions. Scale bars: 20 μm. b) Corresponding time-dependent intracellular fluorescence spectra extracted from the cells over 12 h of incubation. No detectable peak shifts or nonspecific spectral alterations were observed in either control group, validating the structural and photophysical integrity of the individual dye assemblies within cells.

**Figure S6.** Effect of nanoparticle size and cell-type-specific metabolic activity on intracellular dissolution kinetics. a) Representative SEM image of larger PDFNPs fabricated via controlled nucleation and growth. Scale bar: 1 μm. b) DLS profile confirming the monodisperse size distribution of larger PDFNPs centered around 400 nm. c) Fluorescence emission spectra of 100 nm and 400 nm PDFNPs. The identical spectral profiles regardless of dimensions confirm that FRET integrity is maintained in the larger architecture. d) Comparative CLSM images of A549 and HepG2 cells incubated with larger PDFNPs. In A549 cells, the 400 nm-PDFNPs predominantly exhibited F-ON fluorescence (magenta) with negligible F-OFF signals (cyan) even at 12 h, indicating significant resistance to dissolution. In contrast, HepG2 cells exhibited a markedly higher emergence of F-OFF signals, suggesting that the enhanced metabolic machinery of hepatocellular carcinoma cells facilitates more efficient dissolution of large-scale hydrophobic nanoparticles than in lung-derived lineages. Scale bars: 20 μm.

**Figure S7.** Verification of the PDFNP imaging strategy in an immune cell model and tracking of cell-type-specific nanometabolic kinetics. a) Confocal laser scanning microscopy (CLSM) images of RAW264.7 cells incubated with PDFNPs for 3 h and 6 h. Scale bars: 20 μm. b) Corresponding time-dependent intracellular fluorescence spectra extracted from the cells. These results highlight the high sensitivity and versatility of the PDFNP platform in resolving rapid nanometabolic throughput within highly active phagocytic environments.

**Figure S8.** Size-dependent corneal penetration and intratissue nanometabolism of PDFNPs. a) Representative SEM image of large-scale PDFNPs (L-PDFNPs). Scale bar: 1 μm. b) DLS profiles comparing the size distributions of larger PDFNPs (500 nm) and standard PDFNPs (100 nm). c) Fluorescence emission spectra of 100 nm and 500 nm PDFNPs, confirming that FRET integrity and ratiometric optical signatures are preserved regardless of particle dimensions. d) Depth-resolved CLSM images of rat cornea 1 h post-administration of 100 nm PDFNPs. The F-ON signal (magenta) was detected at depths up to 6 μm. In comparison, the F-OFF signal (cyan) emerged from 2 μm onward, indicating that nanoparticle penetration into deep corneal tissue occurs concomitantly with molecular dissolution. e) Comparative CLSM images of rat cornea treated with 500 nm PDFNPs (1 h) and 100 nm PDFNPs (3 h). For the 500 nm L-PDFNPs, significantly attenuated signals were restricted to the superficial epithelium, illustrating the impact of size on tissue-barrier permeability. At 3 h post-administration of 100 nm PDFNPs, the near-complete disappearance of fluorescence, with minimal F-OFF signals remaining at 2–4 μm, signifies rapid intratissue metabolism and clearance of the nanoparticles. Scale bars: 20 μm.

**Figure S9.** Ultrastructural and correlative validation of the membrane-mediated dissolution mechanism. a) Time-resolved emission spectra of PDFNPs across various pH levels, demonstrating exceptional structural integrity in purely acidic aqueous environments. b) Cryo-EM images of the lysosomal mimicry model composed of multilamellar liposomes. The preservation of the phospholipid bilayer architecture after 24 h of continuous stirring confirms that the dissolution is not driven by lipid degradation or micellization. Scale bars: 200 nm. c) Excitation-dependent fluorescence spectra of PDFNPs. The profiles acquired at λex = 355 nm and 470 nm correspond to the cryo-CLEM channel settings. Notably, the 355 nm excitation reveals a discernible peak shift upon dissolution, enabling precise tracking of the metabolic transition. d) Cryo-CLEM images of PDFNPs and the lysosomal mimicry model. In contrast to the intact particulate state, the mimicry model exhibits intense, aggregated F-OFF fluorescence (cyan), validating the localized accumulation and dissolution of PDFNP constituents within the lipidic environment. Scale bars: 20 μm.

**Figure S10.** Cytotoxicity evaluation of PDFNPs across various human cell lines. Cell viability of A549, HeLa, HMEC, and HepG2 cell lines following administration of PDFNPs, as determined by CCK-8 assay (n = 3). PDFNPs exhibited no apparent cytotoxicity across all tested cell lines at a concentration of 0.02 mM, the standard dosage used in the intracellular imaging studies. These results confirm the high biocompatibility of the PDFNP platform, ensuring that the observed nanometabolic transitions occur within physiologically viable cellular environments.
